# Campylobacteriosis Outbreak Associated with Contaminated Municipal Water Supply — Nebraska, 2017

**DOI:** 10.15585/mmwr.mm6807a1

**Published:** 2019-02-22

**Authors:** Caitlin Pedati, Samir Koirala, Tom Safranek, Bryan F. Buss, Anna V. Carlson

**Affiliations:** ^1^Nebraska Department of Health and Human Services, Lincoln, Nebraska; ^2^Division of State and Local Readiness, Office of Public Health Preparedness and Response, CDC.

In March 2017, the Nebraska Department of Health and Human Services (NDHHS) and the Southwest Nebraska Public Health Department were notified of an apparent cluster of *Campylobacter jejuni* infections in city A and initiated an investigation. Overall, 39 cases were investigated, including six confirmed and 33 probable. Untreated, unboiled city A tap water (i.e., well water) was the only exposure significantly associated with illness (odds ratio [OR] = 7.84; 95% confidence interval [CI] = 1.69–36.36). City A is served by four untreated wells and an interconnected distribution system. Onsite investigations identified that a center pivot irrigation system intended to pump livestock wastewater from a nearby concentrated animal feeding operation onto adjacent farmland had malfunctioned, allowing excessive runoff to collect in a road ditch near two wells that supplied water to the city. These wells were promptly removed from service, after which no subsequent cases occurred. This coordinated response rapidly identified an important risk to city A’s municipal water supply and provided the evidence needed to decommission the affected wells, with plans to build a new well to safely serve this community.

## Investigation and Results

On March 10, 2017, NDHHS was notified of five reports of campylobacteriosis in the Southwest Nebraska Public Health Department jurisdiction. Two positive culture reports and three positive culture-independent diagnostic tests, specifically a gastrointestinal polymerase chain reaction (PCR) panel, were received from persons not living together. Campylobacteriosis is a reportable condition in Nebraska, and this number of cases was higher than expected; during 2006–2016, an average of one *Campylobacter* case was reported in a city A resident every 3 years. Initial questioning of ill persons did not include an assessment of exposure to untreated drinking water and suggested ground beef consumption as a possible shared exposure. The Nebraska Department of Agriculture Food Safety and Consumer Protection obtained distribution records for poultry and ground beef for two local restaurants and one local grocery store. The distribution of poultry and ground beef was evaluated by reviewing the routing records of these products to their source, and no evidence of a shared poultry source was identified. The ground beef was not ground in-house at the grocery store, and the distributors that supplied ground beef to the grocery store and each of the two local restaurants were not shared. Through interviews of city A residents and business owners, investigators were made aware of a report of standing water that “smelled of cattle manure” in a roadside ditch near two municipal water wells.

A collaborative on-site investigation revealed that during the pumping of a large volume of livestock wastewater from a concentrated animal feeding operation through a center pivot irrigation system, the system malfunctioned at an undetermined time. The wastewater was intended to be placed on adjacent farmland. This malfunction allowed excessive runoff to flood a road ditch approximately 15 feet (4.6 m) from two municipal water well houses (3 and 4) that had been operating 6 days before the onset of illness in the first patient. The presence of this standing water was confirmed by city A water operators, who reported seeing water in the ditch for 4 days (February 22–25) (Figure). Pump records indicated that during February 22–27, well 3 was in use, and during February 28–March 7, well 4 was in use (Table 1). During both periods, another well (well 2) was also operating. Wells are rotated in and out of service by city operators as part of regular operations. Water is distributed through the well system without any disinfection or filtration. Routine total coliform and *Escherichia coli* testing of water from the distribution system was performed on March 8; however, only wells 2 and 5 were operating on that date. As part of the investigation, additional coliform and *E. coli* testing was performed again on March 16 on direct samples from wells 2, 3, 4, and 5; bacterial culture specifically for *Campylobacter* was performed on March 20 (wells 4 and 5) and 27 (wells 2 and 3). All samples were negative for coliforms and *Campylobacter.* No additional pump or testing records were reviewed.

**FIGURE:**
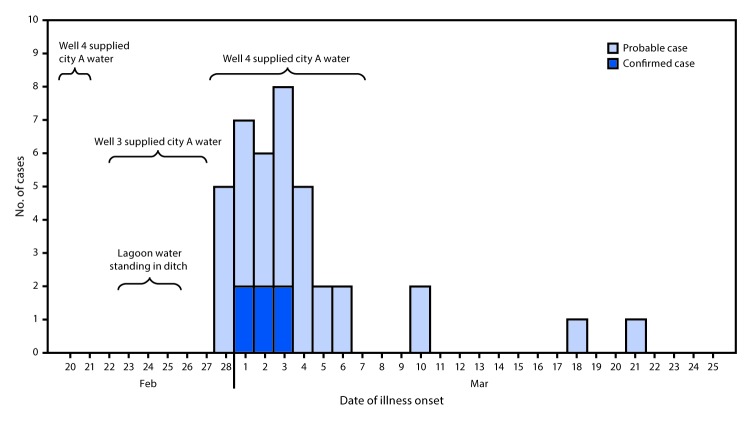
Confirmed (n = 6) and probable (n = 33) campylobacteriosis cases, by date of illness onset and well pumping date — Nebraska, 2017

**TABLE 1:** Volume* and percentage of water pumped from each city A well, by week — Nebraska, 2017

Dates	Volume (%)				Total volume
	Well 2	Well 3^†^	Well 4^†^	Well 5	
February 15–21, 2017	278 (40.0)	0 (0)	417 (60.0)	0 (0)	**695**
February 22–27, 2017	282 (48.8)	296 (51.2)	0 (0)	0 (0)	**578**
February 28–March 7, 2017	327 (40.0)	0 (0)	491 (60.0)	0 (0)	**818**
March 8–13, 2017	207 (32.7)	0 (0)	0 (0)	426 (67.3)	**633**

* x 1,000 gallons.

^†^ Well 3 and well 4 were adjacent to the ditch flooded by livestock wastewater (February 22–25).

On March 16, Nebraska Department of Environmental Quality and the Department of Agriculture conducted an additional investigation of two concentrated animal feeding operation–certified waste lagoons (a manufactured basin that collects livestock waste and water in an oxygen-deprived setting to promote anaerobic conditions as a way to manage refuse)* and associated use of three pivot irrigation systems. The investigation team observed that water from the waste lagoons had been pumped through a pivot onto an adjacent field, which is a common farming practice for fertilizing farm ground or watering crops. City operators confirmed that on February 24 they had observed flow of livestock wastewater into the road ditch near well 4. They followed the wastewater up the road ditch and reported that it came out of the farmland upstream from the wells. Investigators also obtained details of total well depths, static water levels, and pumping water levels (measured during active pumping). Wells 4 and 3 were relatively shallow, with static water levels of 21 and 22 feet, pumping levels of 25 and 26 feet, and total well depths of 43 and 46 feet, respectively; both began service in the 1930s, similar to the other wells in the system, which were also older.

While details around this event were being clarified and environmental testing was pending, an Internet-based questionnaire was designed to aid case-finding and assess potential exposures. A probable case was defined as a diarrheal illness of ≥2 days’ duration with one or more additional signs or symptoms (nausea, vomiting, fever, chills, or headache) in a city A resident, with onset during February 28–March 23, 2017. A confirmed case was defined as a person meeting the probable case definition with either stool culture or PCR-positive results for *Campylobacter*, or a laboratory-confirmed probable illness in a nonresident who worked, dined, or shopped for groceries in city A. Among approximately 600 city A residents, 94 (16%) completed a questionnaire to report food consumption history, drinking water source, animal exposures, and symptoms. Among questionnaire respondents, 39 (41%) campylobacteriosis cases (six confirmed and 33 probable) were identified, with illness onset from February 28–March 21 (Figure); 25 (64%) cases occurred in females and 14 (36%) in males. The median age was 34.5 years (range = 1.5–85 years). Twelve (31%) patients sought medical care, and three (8%) were hospitalized; no deaths were reported.

Data analysis indicated a significant association between ill persons and consumption of untreated, unboiled municipal tap water (OR = 7.84; 95% CI = 1.69–36.36) (Table 2). Other exposures were assessed, including unpasteurized milk, animal contact, raw poultry, and ground beef, but none demonstrated a significant association with illness. Notably, no cases were reported among the approximately 28 residents of city A’s only nursing home, which used city water but treated it with a reverse osmosis system.

**TABLE 2:** Potential exposures reported by survey respondents included for analysis (n = 94) in a community-wide campylobacteriosis investigation and corresponding odds ratios — city A, Nebraska, February 23–March 9, 2017

Exposure	No. (%)				OR (95% CI)*
	Cases (n = 38)		Controls (n = 56)		
	Exposed	Not exposed	Exposed	Not exposed	
City tap water	36 (94.7)	2 (5.3)	39 (69.6)	17 (30.4)	7.84 (1.69–36.36)
Unpasteurized milk	0 (0)	38 (100)	0 (0)	56 (100)	Undefined
Any chicken	12 (33.3)	24 (66.7)	24 (42.8)	32 (57.1)	0.66 (0.27–1.59)
Ground beef	24 (66.7)	12 (33.3)	33 (62.3)	20 (37.7)	1.21 (0.49–2.94)
Animal contact	19 (51.4)	18 (48.7)	40 (72.7)	15 (27.3)	0.39 (0.16–0.95)

**Abbreviations:** CI = confidence interval; OR = odds ratio.

## Public Health Response

Wells 3 and 4 were both permanently removed from service on March 16, and no additional illnesses were reported with onset after March 21. On April 25, NDHHS reclassified these wells to Emergency Status, meaning the well can only be pumped during a case of emergency (e.g., fire, drought, etc.) for nonpotable purposes. Furthermore, meetings were held with area stakeholders to present these findings as evidence to support the award of a planning grant to city A to explore options for a new, higher-volume well to be dug to an acceptable depth in a different location.

## Discussion

This investigation implicates *Campylobacter jejuni* as the cause of this outbreak, most likely from a municipal water system contaminated by wastewater runoff from an adjacent concentrated animal feeding operation (*1*). In addition to environmental and statistical findings, this conclusion is consistent with prior investigations that demonstrate *Campylobacter* outbreaks of similar size are historically associated with contaminated water (*2*–*7*). Although laboratory testing of the water in this investigation did not yield any positive results, samples were not taken until long after the contamination event, and test results might have been affected by switches among wells supplying the system over time. These findings also suggest that routine coliform testing might not be a good indicator of the presence of *Campylobacter* species (*8*). Further, it is possible that *Campylobacter* in particular might be viable but not necessarily detectable by culture in water systems (*9*,*10*). The use of both culture and culture-independent diagnostic tests (PCR) were needed to detect the initial cluster of cases and early recognition of this outbreak. If culture alone had been used, only two cases would have been reported, one of which did not occur in a city A resident. Of those two culture-confirmed cases, one patient refused the interview and the other had typical *Campylobacter* exposures, such as live poultry, which might not have prompted such a rapid response. This investigation demonstrates the importance of considering exposure to untreated water sources as a potential cause for *Campylobacter* outbreaks. Including this risk factor in initial questioning could help to expedite outbreak investigations. Ultimately, early recognition and a coordinated response by several state and local agencies greatly facilitated this successful public health intervention.

**Summary**

**What is already known about this topic?**

*Campylobacter* has been implicated in outbreaks associated with poultry products, unpasteurized milk, and contaminated water sources.

**What is added by this report?**

A center pivot irrigation system intended to pump livestock waste water onto adjacent farmland in Nebraska malfunctioned, allowing excessive run off to collect in a road ditch near two wells that fed a municipal water supply, sickening 39 persons who consumed untreated city water. The use of culture-independent diagnostic tests facilitated case identification allowing for rapid public health response.

**What are the implications for public health practice?**

Access to clean water sources continues to be an important public health issue, and public health professionals should consider exposure to untreated water sources as a potential cause for *Campylobacter* outbreaks.

## Footnotes

* https://www3.epa.gov/npdes/pubs/alagoons.pdf.
